# Forward genetic approaches for elucidation of novel regulators of Lyme arthritis severity

**DOI:** 10.3389/fcimb.2014.00076

**Published:** 2014-06-05

**Authors:** Kenneth K.C. Bramwell, Cory Teuscher, Janis J. Weis

**Affiliations:** ^1^Department of Pathology, University of UtahSalt Lake City, UT, USA; ^2^Department of Medicine, University of VermontBurlington, VT, USA

**Keywords:** Lyme disease, Lyme arthritis, forward genetics, pathogenesis, genome-wide association studies, innate immunity and responses, inflammation, beta-Glucuronidase

## Abstract

Patients experiencing natural infection with *Borrelia burgdorferi* display a spectrum of associated symptoms and severity, strongly implicating the impact of genetically determined host factors in the pathogenesis of Lyme disease. Herein, we provide a summary of the host genetic factors that have been demonstrated to influence the severity and chronicity of Lyme arthritis symptoms, and a review of the resources available, current progress, and added value of a forward genetic approach for identification of novel genetic regulators.

## Introduction to lyme disease

Lyme Disease, caused by infection with the tick borne spirochete *Borrelia burgdorferi*, is a growing societal concern, especially in endemic regions of the United States and Europe. Approximately 30,000 case reports are filed by physicians each year in the United States (C.D.C, 2013a), while the CDC has upwardly revised their best estimate of the total incidence to 300,000/year, based on several complementary lines of evidence (Kuehn, 2013). Part of the societal concern is rooted in the uncertainty surrounding pathological outcomes associated with *B. burgdorferi* infection. A large percentage (70%) of infected individuals develop the characteristic bulls-eye rash *erythema migrans* at the site of the infected tick bite, with progression to further clinical complications following dissemination of the spirochete. Arthritis, the most common symptom occurs in 30–60% of infected individuals, while Bell's palsy and other neurological symptoms are seen in 10–12% of patients (Wormser et al., 2006; C.D.C, 2013b). Carditis has been considered a rare complication (<1%), however, 3 recent deaths with documented *B. burgdorferi* in autopsied heart tissue strongly argue for increased vigilance in detecting infection of this tissue (C.D.C, 2013c).

The wide variation in Lyme disease symptoms and severity observed within the patient population is thought to reflect unique features of individual *B. burgdorferi* isolates that influence invasive potential, as well as heritable factors in the patient population that contribute to clinical severity. Furthermore, although most patients resolve infection with appropriate antibiotic therapy, a small percentage of treated patients with severe clinical symptoms fail to resolve and develop a chronic disease termed Post Treatment Lyme Disease (Steere and Glickstein, 2004). Thus, there are compelling reasons to identify host genes that determine the severity of Lyme disease, both in understanding the pathogenic mechanisms of acute clinical disease and in characterizing predisposing features for chronic disease. This review will assess past and ongoing studies that have provided insight into genetic susceptibility to Lyme arthritis, with particular emphasis on studies using Forward Genetic approaches.

## What is forward genetics?

Forward genetics is an unbiased genetic approach that begins with a heritable trait of interest and attempts to determine the alleles responsible for the observed variability through a process of genetic mapping. In contrast, reverse genetics is a hypothesis-driven scientific approach that begins with a gene of interest and attempts to determine the phenotypic impacts caused by experimental manipulations of that gene. Classically, forward genetic studies were first performed through random mutagenesis screens. More recently, forward genetics has been used to map pre-existing genetic variations in human populations or experimental animal models. In practice, forward genetic studies involve three key steps: (1) Each individual in a population of mixed genetic composition is surveyed for the trait of interest; (2) The genetic makeup of each individual is assessed; (3) Statistical calculations predict the strength of association in the study population between the measured trait and each genetic locus in the genome. Forward genetic screens often produce a map across the entire genome with peaks and valleys denoting areas with strong or weak statistical association with the trait, respectively.

Scientific investigations into heritable genetic risk factors that contribute to complex disease have been conducted using a variety of approaches, including Genome Wide Association Studies (GWAS) and forward genetic screens in tractable animal models.

## Genome wide association studies as a methodology for studying complex genetic traits in humans - successes and limitations

Genome Wide Association Studies were first proposed in the mid-1990s as a way to study association between human genetic polymorphisms and complex, multigenic traits. Rather than measure all genetic variation present in each individual, approximately one million Single Nucleotide Polymorphisms (SNPs) are assayed as genetic landmarks, and scored for association with the trait of interest. Based on linkage disequilibrium, genes in close proximity to associated SNP landmarks are potential candidates for further investigation.

This technique is well-suited to identify susceptibility genes that are of intermediate prevalence in the population, with a Mean Allele Frequency of greater than 0.05 (Risch and Merikangas, 1996). Although no such studies of Lyme arthritis severity have been conducted, GWAS has been extensively used to investigate genetic modulators of other inflammatory conditions including rheumatoid arthritis (RA). Thus far, almost fifty susceptibility loci have been identified for RA, accounting for approximately one-half of the total genetic variation expected in populations of European ancestry. Nineteen of these loci have been refined to a single candidate gene association, and the underlying causal polymorphism has been predicted for seven of these loci (Eyre et al., 2012).

Recent studies with RA and juvenile RA have involved cohorts of up to 10,000 patients and controls, pointing out the requirement for large populations of well-characterized patients for GWAS analysis (Hinks et al., 2013). If sufficiently large sample sizes could be achieved, these findings suggest that GWAS could be a successful strategy to investigate Lyme arthritis susceptibility loci, but also indicate that additional approaches are needed to capture the significant fraction of variation likely to be left unaccounted for.

It is also important to recognize that identification of regulatory loci is not the ultimate goal of a forward genetics study, but is only a first step. While association of a specific genetic landmark to disease susceptibility may have potential relevance to clinical diagnosis, there is added value in the formal investigation of candidate genes and predicted causal polymorphisms, and in further understanding the underlying mechanisms of pathogenesis. This type of mechanistic investigation frequently involves the use of animal models.

## Use of animal models for identification of genes regulating disease severity

Animal models provide an alternative approach for identification of genes that regulate disease development. Inbred mouse lines are powerful genetic resources, which have been widely used to identify genes associated with disease severity. Visionary scientists began the breeding of inbred mice over a century ago. Each modern inbred line has fixed genetic composition, while the plentitude of inbred strains collectively capture a large amount of genetic variation. Several advances in the past decade have significantly added to their value, particularly the publication of the mouse genome, coupled with various efforts to define the genetic variation between inbred mouse strains (Gregory et al., 2002; Keane et al., 2011). More recently, the Collaborative Cross was developed, which represents an ambitious community effort by mouse geneticists to develop approximately 1000 additional recombinant inbred mouse strains with defined genetic composition. These recombinant mice were derived from 8 parental inbred and wild-derived strains through an intricate directed breeding process (Churchill et al., 2004). Due to the increased genetic diversity of mouse strains that can be interrogated simultaneously, the Collaborative Cross is expected to provide additional power to forward genetics screens for many diseases. Together, these more recent developments are expected to provide greater predictive power for identification of regulatory intervals underlying complex multigenic traits.

Prior to the availability of these more sophisticated modern resources, a seminal study by Dr. Stephen Barthold recognized that inbred mouse strains exhibit distinct genetic susceptibilities to Lyme arthritis, recapitulating the range of arthritis severity seen in patients (Barthold et al., 1990). However, mice do not recapitulate the full depth and breadth of symptoms experienced by human patients. This is evident in the inability of *B. burgdorferi* to elicit neurological symptoms or *erythema migrans* in mice (Garcia-Monco and Benach, 2013). Despite these limitations, the finding that C3H mice develop severe arthritis and carditis at reproducible times following intradermal infection with *B. burgdorferi* cultured in the laboratory was very important. The C3H mouse has been used extensively for studies of severe disease, and the involvement of a variety of cell types and signaling pathways have been evaluated. Equally important was the observation that C57BL/6 (B6) mice consistently develop less severe disease despite being equally susceptible to infection and having similar numbers of bacteria in joints. Since many mutant alleles have been crossed onto the B6 background, this has allowed identification of the contribution of numerous immunologically important genes to both host defense and modulation of arthritis and carditis severity.

Mice and other small mammals are essential reservoir species for *B. burgdorferi* in nature, and greater than 90% of trapped wild mice in some Lyme endemic areas have tested seropositive for infection (Bunikis et al., 2004; Radolf et al., 2012). Wild mice are generally resistant to Lyme arthritis, although recent work has shown that natural variants of known innate immune regulatory genes may be correlated with the prevalence of Lyme infection within the wild rodent population (Tschirren et al., 2013). These considerations make the mouse an excellent model for assessment of genetic factors contributing to Lyme arthritis development.

Barthold's findings were corroborated and expanded upon by others. Many groups then addressed specific facets of the immune response and pathogenesis of Lyme arthritis, using the mouse models developed by Barthold, and that important work continues today. Many such studies rely on reverse-genetic approaches, such as targeted genetic deletion, gene silencing, treatment with inhibitory or stimulatory molecules, or transgenic manipulation. For example, the importance of the innate immune response in Lyme pathogenesis was demonstrated by Schaible, Barthold, and Brown who collectively observed that mice with severe combined immunodeficiency (*scid/Rag*^−^), lacking B and T cells, retained the differential genetic severities in arthritis and carditis observed between inbred mouse strains (Schaible et al., 1989; Barthold et al., 1992; Brown and Reiner, 1999). This finding set a lasting framework for future studies into various facets of the innate immune response. The reverse genetic techniques used in these and other studies are powerful and conclusive, and have resulted in the identification of many genes with documented importance in the pathogenesis of Lyme arthritis and host defense to *B. burgdorferi*. However, these approaches are by nature biased to genes with known function and are not suitable for global analysis of the potential genetic contribution to disease. Selection of a candidate gene necessarily involves assessment of pathways that are suspected to influence the disease process, resulting in the rejection or delay of other non-candidate genes for study.

## Brief review of genetic associations with human lyme disease and related mouse studies

### Role of the MHC in immune response to *B. burgdorferi* and in arthritis severity: human and mouse studies

Several important studies have discovered natural genetic alleles that influence Lyme arthritis severity. Steere et al. first reported the influence of the human major histocompatibility complex (MHC) on Lyme arthritis severity, and provided important early evidence that Lyme arthritis has an immunogenetic basis (Steere et al., 1990). This pioneering work identified increased incidence of clinical Lyme arthritis, particularly that lasting longer that 12 months in a single joint, as associated with two serologically defined Class II alleles, HLA-DR4 and HLA-DR2. Importantly, the association of Class II alleles with Lyme arthritis was not supported by studies inclusive of all outcomes of Lyme arthritis patients. The advent of molecular characterization of Class II alleles allowed more precise analysis of associations with disease phenotype, and led to the conclusion that MHC alleles are not major determinants of early Lyme disease severity, a distinction from rheumatoid arthritis (Feng et al., 1995; Klempner et al., 2005). More recently, Steere and colleagues have confirmed the association of two Class II alleles (DRB1^*^0101 and 0401) for the subgroup of patients with treatment refractory Lyme disease but not in the larger group of patients that respond to antibiotic treatment, and have proposed an auto-immune mechanism in this treatment refractory group (Steere et al., 2006; Drouin et al., 2013).

A number of investigators found association of MHC haplotypes with antibody recognition of individual *B. burgdorferi* antigens using MHC congenic mouse lines. However, use of MHC congenics in our studies and in those of other investigators led to the conclusion that MHC alleles were not determinants for the differences in arthritis severity found 4 weeks following infection in C3H-*H2^k^*, C57BL/6-*H2^b^*, and DBA-*H2^d^* mice (Yang et al., 1992; Brown and Reiner, 2000). Thus, studies with mice are consistent with patient studies failing to show association with early Lyme arthritis. Interestingly, mice expressing the *H2^k^* allele do not develop collagen-induced arthritis, a contrast with their development of severe Lyme arthritis (Wooley et al., 1981).

### Identification of TLR1/TLR2 in the host response to *B. burgdorferi* in humans and mice

Early seminal studies into the host-pathogen interaction of *B. burgdorferi* revealed the potential of the spirochete and its lipoproteins to induce inflammatory cytokine production in a variety of human and mouse cell types (Radolf et al., 1991; Wooten et al., 1996; Sellati et al., 1998). The association of NF-κB with these inflammatory responses directed numerous laboratories to investigate the involvement of Toll-like receptors as these molecules were discovered as central components of inflammatory responses to microbial pathogens (Wooten et al., 1996; Sellati et al., 1998). These studies documented the interactions between *B. burgdorferi* lipoproteins with TLR2 and TLR1, both with mouse knock-out and cell culture transfection studies and in patients, and established a critical role for TLR signaling through MyD88 in host defense to this pathogen (Aliprantis et al., 1999; Brightbill et al., 1999; Hirschfeld et al., 1999, 2000; Alexopoulou et al., 2002). More recent studies by Schroder et al. identified a human variant in TLR2, Arg753Gln, with reduced pro-inflammatory signaling in patient samples (Schroder et al., 2005). Cells from mice heterozygous for this variant also displayed reduced inflammatory responses to *B. burgdorferi* lysate. Notably, this TLR2 allele was significantly underrepresented within a cohort of late stage Lyme disease patients, suggesting that it has a protective effect.

Oosting et al. found that N248S and S602I polymorphisms in TLR1 were associated with reduced *in vitro* responsiveness to *B. burgdorferi* and TLR1/TLR2 agonist stimulation (Oosting et al., 2011a). Using a similar experimental approach, the same group also reported that peripheral blood mononuclear cells (PBMCs) for individuals bearing an IL-23R Arg381Gln polymorphism exhibited a reduced Th17 response following *in vitro* stimulation with *B. burgdorferi* (Oosting et al., 2011b). However, there was no association between the IL-23R polymorphism and the persistence of symptoms among patients in the study population, arguing against a role for this SNP in disease pathogenesis.

Strle et al. recently described the frequency and impact of several polymorphisms in the TLR1 gene within a cohort of Lyme disease patients (Strle et al., 2012). This study found a skewed inheritance pattern of TLR1 1805GG polymorphisms within an antibiotic-refractory Lyme arthritis patient population. They also recognized a synergy between inheritance of this host polymorphism and infection with a particular invasive isolate (termed RST1) of *B. burgdorferi*. Importantly, patients carrying TLR1 1805GG exhibited higher serum levels of CXCL9 and CXCL10 chemokines, consistent with a functional role for this polymorphism. This effect was reproduced through *in vitro* activation of PBMCs with a *B. burgdorferi* RST1 isolate, arguing that heightened production of these IFNγ-inducible chemokines may set the stage for antibiotic refractory arthritis.

## The power of forward genetics

Forward Genetic approaches attempt to determine which genetic loci are responsible for a phenotype of interest. In general, individuals are generated with genotypes that have been altered in an unbiased way, followed by analysis to map inheritance of the phenotype of interest to specific genetic loci. This was made possible by the development of genetic maps of microsatellite landmarks evenly distributed throughout the genome, the utility of which Paterson et al. first demonstrated for Quantitative Trait Locus (QTL) mapping in plants, later followed by Todd et al. in mice (Paterson et al., 1988; Todd et al., 1991). Model organisms are often studied through QTL analysis followed by the breeding of recombinant inbred congenic lines to isolate regulatory loci, and the Collaborative Cross is a more expansive modern variation of this theme that combines these two steps together. QTL mapping of disease susceptibility in mice has the potential to yield a veritable avalanche of information about complementary facets of disease initiation and pathogenesis. For example, efforts by Edward Wakeland and others to determine differential susceptibility to systemic lupus erythematosus (*sle*) between resistant and acutely lupus-prone inbred mouse strains led to the identification of *Ly108* and other SLAM family members as key modulators of B cell tolerance (Kumar et al., 2006), lack of proper *Fcgr2b* upregulation as a potentiator of IgG production (Rahman et al., 2007), *Cr2* or other closely linked genes as mediators of autoreactive B- and T-cell production (Chen et al., 2005; Tchepeleva et al., 2010), and hemostatic *kallikreins* as important regulators of kidney pathogenesis (Liu et al., 2009).

Other successes include the identification of genes important in the regulation of animal models of rheumatoid arthritis and autoimmunity by comparing disease susceptible and disease resistant mouse strains (Ma et al., 2002; Glant et al., 2004; Wicker et al., 2005; Ahlqvist et al., 2009). In some cases, QTL mapping efforts have bridged gaps between seemingly distinct experimental models of autoimmune and other inflammatory diseases through the identification of shared immunopathology loci (Teuscher, 1985; Meeker et al., 1995; Teuscher et al., 1996, 1997, 1998; Del Rio et al., 2008; Spach et al., 2009, 2010) and identification of the relevant functional polymorphisms (Sudweeks et al., 1993; Ma et al., 2002).

**Figure 1:**
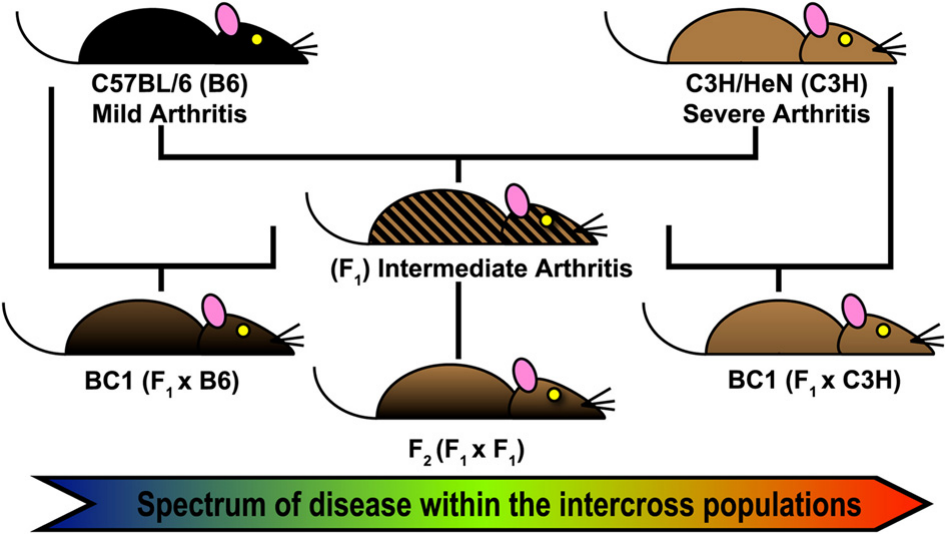
**Forward Genetics approach for Lyme arthritis severity**. Intercross populations of B6 and C3H mice were used for identification of Quantitative Trait Loci (QTL) regulating *Borrelia burgdorferi* associated arthritis and other responses related to infection (*Bbaa*). Arthritis and other metrics of host response were assessed at 4 weeks of infection. A total genome scan was performed for each infected mouse (*n* = 450) and threshold permutation analysis identified loci associated with disease and response.

The first step of QTL analysis in mice is the direct interbreeding of two strains of interest to generate a large cohort of genetically distinct individuals (Figure 1). F_1_ hybrids are genetically identical, carrying one copy of each chromosome from each parental line. These hybrids can be backcrossed to either parental strain (BC1), or interbred to generate F_2_ hybrids. In each case, genetic variability among the offspring is generated by random recombination events between sister chromatids during meiosis. Each interbreeding strategy can identify regulatory alleles with a dominant, codominant, or additive effect. F_2_ intercross populations allow the identification of alleles acting in recessive fashion that are capable of “standing alone,” whereas the BC1 populations have the added advantage of allowing identification of genetic alleles whose effect is most apparent in the genetic context of a particular inbred background. However, hybrids backcrossed to a parental strain are not expected to detect any phenotype from recessive alleles bred back to a dominant parent.

## Current progress: mapping quantitative trait loci that regulate lyme arthritis severity in mice

Janis Weis, Cory Teuscher, and their collaborators performed the first murine Lyme arthritis QTL analysis (Weis et al., 1999). This initial study used an F_2_ intercross between C57BL/6N (B6) and C3H/HeN (C3H) mice, with 150 total male and female mice included in the cohort. Each individual was assessed for seven quantitative traits and for genetic composition, which was determined using 195 microsatellite markers distributed throughout the genome. Permutation threshold analysis was then used for the entire cohort to determine the degree of association between these quantitative traits and the parental derivation of specific loci. Four distinct regions on chromosomes 4, 5, and 11 were found to regulate arthritis severity traits as measured by caliper measurement of ankle swelling and by blinded scoring of a number of microscopically assessed histopathology traits. Five additional loci on chromosomes 6, 9, 11, 12, and 17 were found to regulate *B. burgdorferi*-specific humoral IgM and IgG responses independently of arthritis severity.

**Figure 2:**
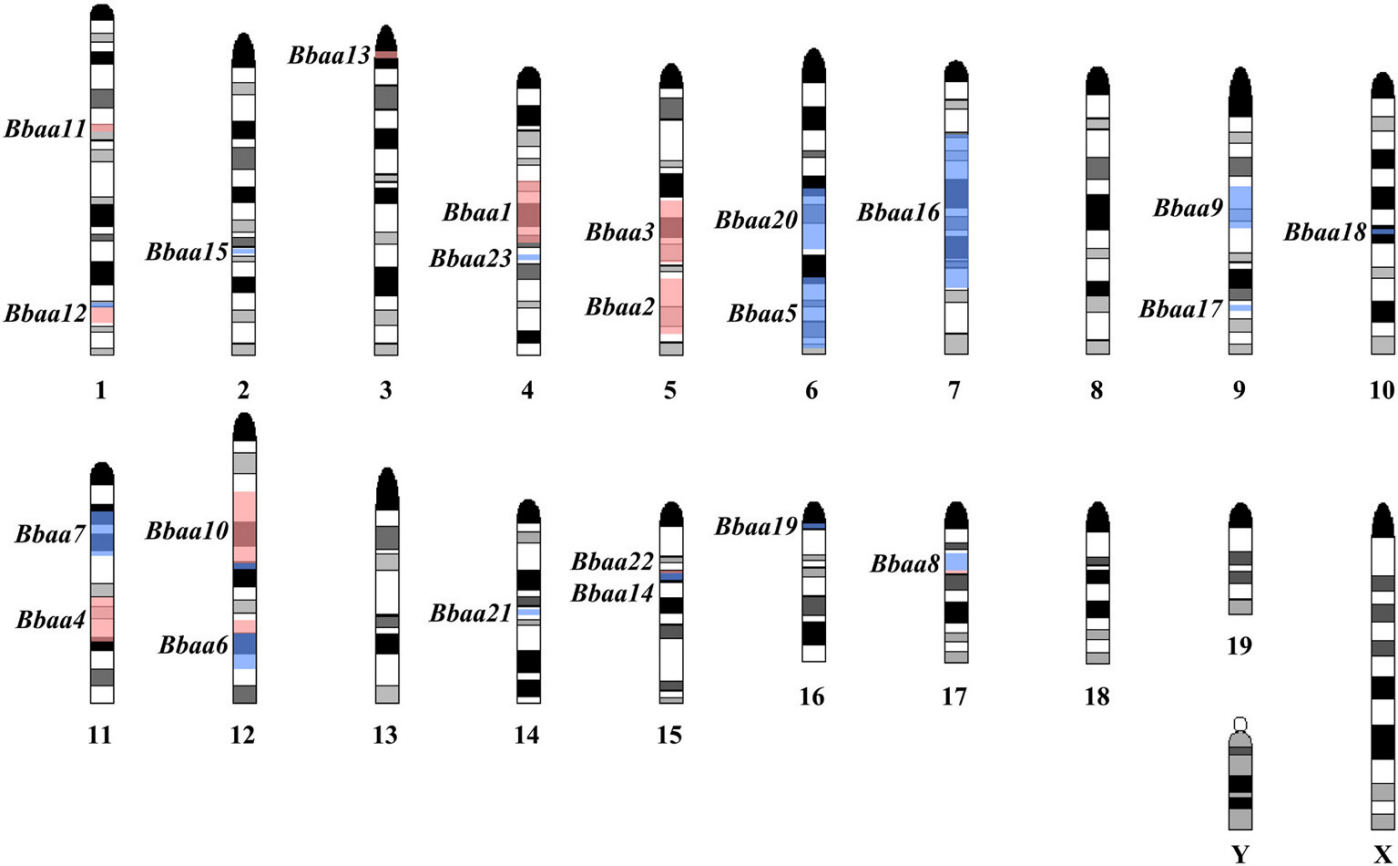
**Location of predicted Quantitative Trait Loci, summarized from Weis et al. (1999) and Roper et al. (2001)**. Black and gray bands on each chromosome denote an idealized representation of the Giemsa banding pattern found in a normal mouse karyotype. Red shaded regions denote QTL regulating arthritis severity (ankle swelling, histopathology score, tendon sheath). Blue shaded regions denote QTL regulating humoral immune response (Total/Specific IgM levels, Total/Specific IgG levels, serum IL-6) or host defense (*B. burgdorferi* bacterial burden).

This foundational study was followed up by Roper et al. with additional QTL experiments using reciprocal F_1_ × B6 and F_1_ × C3H backcrosses and a (BALB/c × C3H) F_1_ × C3H intercross that found 12 new QTL on Chromosomes 1, 2, 4, 6, 7, 9, 10, 12, 14, 15, 16, and 17 regulating a variety of traits (Roper et al., 2001). A total of twenty-three QTL were identified that regulate metrics of arthritis severity (Figure 2, red) or other traits related to the humoral response, inflammatory response, or host defense (Figure 2, blue). As predicted from previous MHC congenic studies, none of the arthritis-associated QTL identified in the three B6:C3H intercrosses identified the MHC locus on chromosome 17. Interestingly, ankle swelling did associate with this region in a single backcross (BALB/c × C3H) F_1_ × BALB/c, with a lod score of 3.1, predicting an association with one or more of the numerous class I, class II, or class III genes in this region. Seven of the 23 QTL were reproduced in multiple crosses (*Bbaa2, Bbaa6, Bbaa8, Bbaa10, Bbaa12, Bbaa14*, and *Bbaa15*). The *Bbaa2* QTL on Chromosome 5 was reproduced in all four intercross experiments, and in every case the arthritis severity originated from the C3H parental strain. The lod scores identifying *Bbaa2* ranged from 3.5 to 10.2 for the four intercross populations, with the 10.2 lod value detected in the (BALB/c × C3H) F_1_ × C3H intercross. This study also predicted that the combined *Bbaa2Bbaa3* locus contains at least four distinct regulatory genes. It is possible that some of these loci may be implicated in other QTL studies, but the use of different inbred strains of mice and the extensive polymorphism of this region of the genome among strains confounds the ability to directly extrapolate between studies (Lindvall et al., 2009).

Based on these and other data, several subsequent studies generated congenic mouse strains to isolate putative regulatory loci in the context of an otherwise uniform genetic background. This laborious and time-intensive process is essential to convert the statistically predicted loci derived from QTL analysis into physical genetic boundaries. Congenic lines can also be used to formally interrogate potential candidate genes, by determining if the phenotype of interest is retained after such a candidate gene is excluded from the congenic interval. The presence of a strongly penetrant phenotype within a congenic interval is a strong predictor of success in further steps of positional cloning.

Subsequent studies used congenic mice based on the initial QTL assignments. Crandall et al. described the phenotype of two B6 × C3H congenic lines, and the evaluation of a specific candidate gene (Crandall et al., 2005). B6 × C3H F_1_ mice were backcrossed seven times onto each parental background, producing reciprocal congenic lines B6.C3H-*Bbaa2Bbaa3* and C3H.B6-*Bbaa2Bbaa3*. Of note, the congenic nomenclature in mice differs from other systems, with the background strain listed first, followed by the donor strain, followed by the introgressed locus (Jackson Laboratory, 2000). *Bbaa2Bbaa3* from the C3H donor strain was found to confer increased Lyme arthritis severity on a resistant B6 background, while B6 derived *Bbaa2Bbaa3* conferred reduced severity to susceptible C3H mice, in a reciprocal fashion. This publication also described a polymorphism carried by C3H mice in the *Ncf1* gene, but ruled out this candidate with a variety of studies, including the finding that B6 *Ncf1*^−/−^ mice exhibited no increase in arthritis severity relative to wild type B6 controls.

Ma et al. reported the generation of additional B6xC3H reciprocal congenic lines for five intervals identified in the foundational QTL study (*Bbaa1, Bbaa2Bbaa3, Bbaa4*, and *Bbaa6*), plus another pair of reciprocal congenic lines for an interval on Chromosome 1 (*Bbaa12*) (Ma et al., 2009). Through marker-assisted selection over the course of seven iterative backcrosses, these intervals were isolated and found to be free of genetic contamination on other chromosomes, with congenic intervals ranging from 25 to 146 megabases in size. *Bbaa2Bbaa3* and *Bbaa4* were found to reciprocally transfer the ankle swelling and histopathology phenotypes, while the B6 allele of *Bbaa6* transferred protection from ankle swelling and histopathology to the C3H background. Other congenic intervals conferred no change in arthritis severity or gave inconsistent results. This study also demonstrated the added utility of congenic lines as an experimental resource through comparative microarray gene expression profiling.

**Figure 3:**
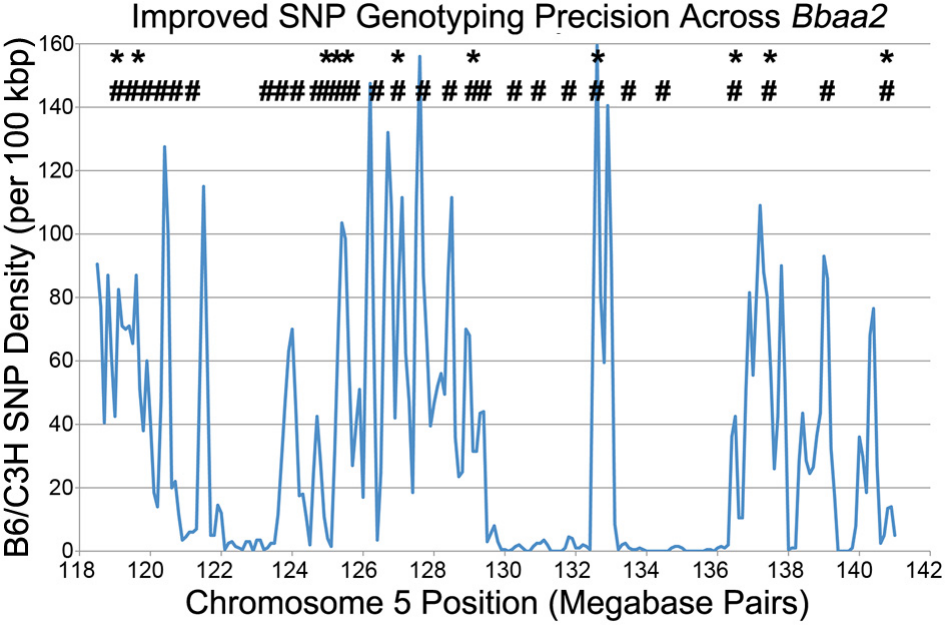
**A SNP genotyping methodology improves congenic mapping precision, adapted from Bramwell et al. (2012)**. ^*^ - Location of all 11 microsatellite markers that can differentiate between B6 and C3H DNA across *Bbaa2*. **#** - Of the thousands of SNPs available (blue line), the 28 positions that were developed into SNP genotyping assays for improved discrimination of sub-interval congenic lines (Bramwell et al., 2014).

In the process of further refining these congenic intervals, Bramwell et al. described the implementation of high throughput SNP genotyping and high resolution melting analysis (Bramwell et al., 2012). This improved genotyping methodology took advantage of the recently published Sanger high-resolution sequence of the C3H mouse, allowing enhanced comparison with the previously published genome of the B6 reference strain (Keane et al., 2011). The Sanger database revealed the precise location of thousands of SNPs distinguishing B6 and C3H genomic sequences within the 20 Mbp *Bbaa2* interval, exponentially increasing the ability to discriminate donor sequences and define boundaries of congenic mice, and allowing the genetic composition of the congenic lines to be tested with greater precision (Figure 3). As an added benefit, the screening process was accelerated, helping to reduce expenses by allowing litters to be screened prior to weaning age.

**Figure 4:**
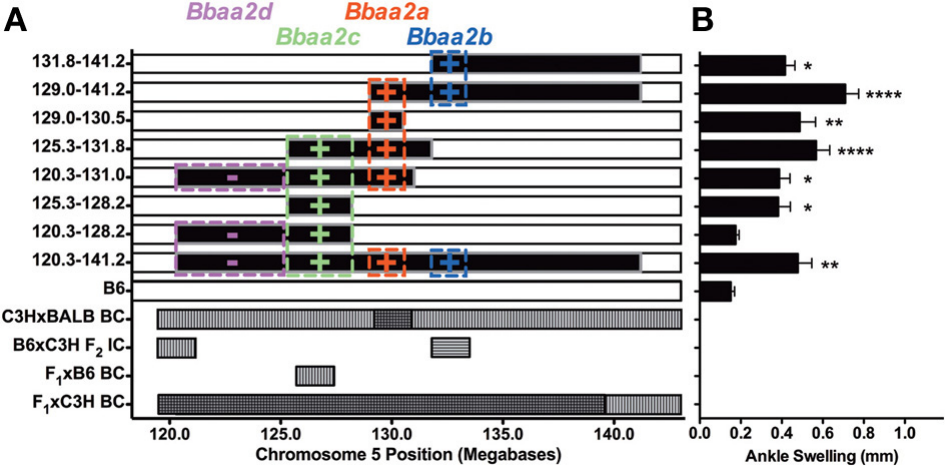
**Regulatory loci identified by analysis of advanced congenic lines, modified from Bramwell et al. (2014)**. **(A)** Each of the top 8 horizontal bars represents one B6.C3H-*Bbaa2* sub-interval congenic mouse line. The horizontal axis represents the position of *Bbaa2* on mouse Chromosome 5 (120.3–141.2 Mb). The black portions of each row are derived from the C3H genetic background and the white portions are derived from the B6 background. Colored boxes indicate the position boundaries and predicted effect (+/−) of multiple regulatory intervals identified by advanced congenic lines. The lower 4 rows indicate arthritis severity QTL intervals predicted by Roper et al. (2001) for the backcross/intercross populations listed on the left vertical axis. Horizontal hatching denotes QTL for ankle swelling, vertical hatching denotes QTL for histopathology or tendon sheath thickening, cross hatching denotes overlap of multiple predicted QTL. **(B)** Ankle swelling measurements for the eight congenic lines listed in A, with significance assessed relative to B6 negative control. ^*^P < 0.05, ^**^P < 0.01, ^****^P < 0.0001.

**Figure 5:**
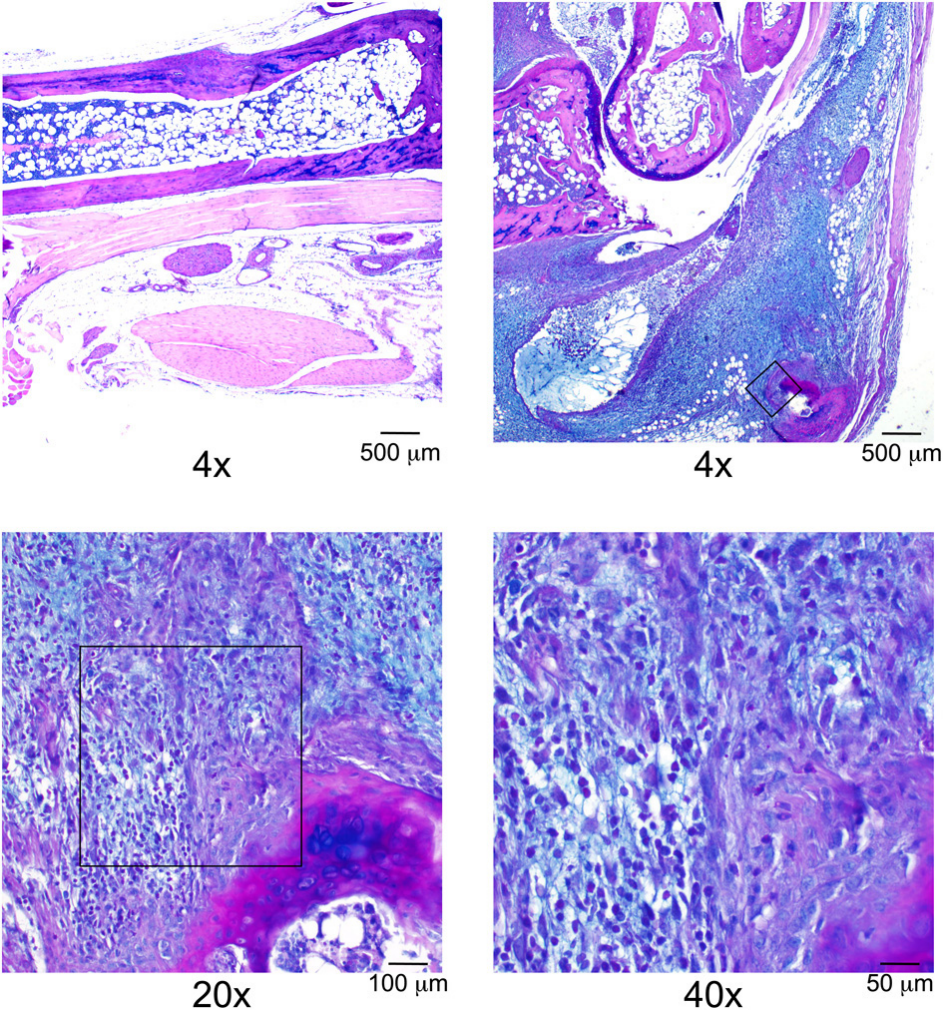
**Alcian blue staining reveals excess deposition of GAGs within severely arthritic ankle joints, modified from Bramwell et al. (2014)**. Top left panel: Ankle joint section from a day 7 K/BxN treated B6 mouse at ×4 magnification. Remaining panels: Ankle joint section from a day 7 K/BxN treated B6.C3H-*Gusb^h^* mouse. Original magnification ×4, ×20, and ×40. Scale bars: 500, 100, 50 μm, respectively. Boxes on the top right and lower left images indicate the location of the field magnified in subsequent images.

This line of investigation has recently culminated in the identification of the first definitive natural regulator of Lyme arthritis severity in laboratory mice. With further backcrossing and refinement of the B6.C3H-*Bbaa2* congenics, Bramwell et al. describe the generation of 14 new advanced congenics that delimit the boundaries of several regulatory sub-intervals (Figure 4) (Bramwell et al., 2014). Notably, these intervals bear striking resemblance to several of the maximal linkage peaks predicted previously (Roper et al., 2001). One narrow 1.5 Mb C3H-derived interval, surrounding and including the highest peak of linkage predicted by QTL analysis at D5Mit30 on Chromosome 5, was able to independently confer an increased arthritis severity phenotype in the context of a resistant B6 genetic background. Close scrutiny of this interval revealed only a single coding-non-synonymous polymorphism between B6 and C3H mice. This point mutation in the lysosomal enzyme beta-Glucuronidase leads to a partially hypomorphic allele (*Gusb^h^*) in the C3H, AKR, and CBA/J inbred strains. *Peromyscus* mice, which do not exhibit Lyme arthritis but serve as important reservoir hosts for *B. burgdorferi* in nature, appear to carry the wild-type B6 allele of *Gusb* (GenBank Accession XM_006971357). The exacerbated Lyme arthritis effect conferred by *Gusb^h^* was recapitulated in a spontaneous *Gusb* mutant mouse line (*Gusb^Null^*), and transgenic overexpression of wild type *Gusb^b^* in C3H mice (*Gusb^Tg^*) profoundly reduced ankle swelling and histopathology. The *Gusb^h^* congenic line was further tested in an experimental model of RA, the K/BxN serum transfer model (Monach et al., 2008). Disease severity in this model is induced by autoantibodies generated against glucose-6-phosphate isomerase, a ubiquitous glycolytic enzyme. Importantly, transfer of this serum induces a joint specific inflammatory arthritis that occurs independently of the MHC haplotype of the recipient and reflects the effector phase of arthritis development. Much greater arthritis severity was observed in *Gusb^h^* congenic mice than in wild type B6 control animals, revealing a common mechanism for the pathogenesis of Lyme arthritis and rheumatoid arthritis. Thus, the identification of genes important in Lyme arthritis also illuminated previously unrecognized pathways in RA. This linkage to a gene associated with Sly syndrome, an overt congenital lysosomal storage disease (LSD), strongly implicated a common pathogenic mechanism involving accumulation of undigested glycosaminoglycans (Tomatsu et al., 2009). This possibility was confirmed by detection of pronounced Alcian blue staining of sulfated GAGs in the inflamed joint tissues of *B. burgdorferi* infected and K/BxN treated mice with partial or severe *Gusb* deficiencies (Figure 5). The association of *Gusb^h^* with increased disease severity in both Lyme-associated and rheumatoid arthritis identifies *Gusb* as a shared immunopathology disease gene (Teuscher, 1985; Sudweeks et al., 1993; Ma et al., 2002).

The novelty of the beta-Glucuronidase polymorphism highlights the power and added value of forward genetic approaches. *Gusb* is most often cited in the recent scientific literature as a housekeeping gene, primarily used as a reference to study something more interesting, making it a most unlikely candidate for a hypothesis-driven reverse genetics study. Allelic variants of the *Gusb* gene were found not to be differentially expressed under baseline conditions, and no changes in *Gusb* expression were detected by microarray analysis of joint tissue from naïve and infected C3H and B6 mice (Crandall et al., 2006; Bramwell et al., 2014). Thus, *Gusb* and other similar genes associated with LSD are not likely to be picked up by a microarray or RNA-Seq study in Lyme arthritis patients. *Gusb* was also not included in the ImmunoChip used in human RA and juvenile RA studies, because it had not yet been identified as a potential regulator (Eyre et al., 2012; Hinks et al., 2013). As mentioned earlier, recent development of an expanded group of recombinant inbred strains incorporating 8 strains of laboratory and wild mice would appear to be a powerful resource for investigating regulators of Lyme arthritis severity. However, none of the three inbred strains carrying the *Gusb^h^* polymorphism were included in the Collaborative Cross, so it could not have been identified through this approach. Against all odds, the identification of *Gusb* is a prime example of how relentlessly following the phenotype throughout a process of unbiased genetic refinement can overcome preconception and bias to lead the way to truly novel and unexpected discoveries.

### Conflict of interest statement

The authors declare that the research was conducted in the absence of any commercial or financial relationships that could be construed as a potential conflict of interest.
